# RNA-Based TWIST1 Inhibition via Dendrimer Complex to Reduce Breast Cancer Cell Metastasis

**DOI:** 10.1155/2015/382745

**Published:** 2015-02-11

**Authors:** James Finlay, Cai M. Roberts, Gina Lowe, Joana Loeza, John J. Rossi, Carlotta A. Glackin

**Affiliations:** ^1^Department of Neurosciences, City of Hope Beckman Research Institute, 1500 East Duarte Road, Duarte, CA 91010, USA; ^2^Irell & Manella Graduate School of Biological Sciences, City of Hope Beckman Research Institute, 1500 East Duarte Road, Duarte, CA 91010, USA; ^3^Division of Comparative Medicine, City of Hope Beckman Research Institute, 1500 East Duarte Road, Duarte, CA 91010, USA; ^4^Department of Biological Sciences, California State University, Los Angeles, 5151 State University Drive, Los Angeles, CA 90032-8201, USA

## Abstract

Breast cancer is the leading cause of cancer-related deaths among women in the United States, and survival rates are lower for patients with metastases and/or triple-negative breast cancer (TNBC; ER, PR, and Her2 negative). Understanding the mechanisms of cancer metastasis is therefore crucial to identify new therapeutic targets and develop novel treatments to improve patient outcomes. A potential target is the TWIST1 transcription factor, which is often overexpressed in aggressive breast cancers and is a master regulator of cellular migration through epithelial-mesenchymal transition (EMT). Here, we demonstrate an siRNA-based TWIST1 silencing approach with delivery using a modified poly(amidoamine) (PAMAM) dendrimer. Our results demonstrate that SUM1315 TNBC cells efficiently take up PAMAM-siRNA complexes, leading to significant knockdown of TWIST1 and EMT-related target genes. Knockdown lasts up to one week after transfection and leads to a reduction in migration and invasion, as determined by wound healing and transwell assays. Furthermore, we demonstrate that PAMAM dendrimers can deliver siRNA to xenograft orthotopic tumors and siRNA remains in the tumor for at least four hours after treatment. These results suggest that further development of dendrimer-based delivery of siRNA for TWIST1 silencing may lead to a valuable adjunctive therapy for patients with TNBC.

## 1. Introduction

Breast cancer is the leading cause of cancer-related deaths among women in the United States with over 235,000 new diagnoses and roughly 40,000 deaths expected in 2014 [1]. Nonmetastatic breast cancer is relatively well-managed with chemotherapy, radiation, and surgery. However, metastatic breast cancer (MBC) that has spread to the liver, bone, brain, and lungs is frequently incurable [2, 3]. Triple-negative breast cancer (TNBC) (ER-negative, progesterone receptor (PR) negative, and HER2-negative) is of particular interest, because it is aggressive and metastatic and does not respond to current therapies. Understanding the metastatic mechanisms of the aberrant cancer cells that allow them to spread to distant sites in the body and develop into metastatic tumors is therefore crucial, in order to identify new therapeutic targets and develop novel treatments that can be used in conjunction with current therapies to prevent metastases and improve patient outcomes.

A key mechanism for the spread of cancer cells is epithelial-mesenchymal transition (EMT). During EMT, cancer cells undergo changes enabling them to detach from the primary tumor and invade into surrounding tissues, the lymphatic system, and blood vessels [4–7]. EMT also allows mobile cancer cells to migrate out of blood vessels and into distant organs. The TWIST1 transcription factor activates EMT in cancer cells [8, 9] and activates several target genes that promote cellular dedifferentiation and cell mobility. In addition to promoting EMT in cancer cells, TWIST1 is thought to promote the cancer stem cell (CSC) phenotype [10], inhibit apoptosis [11, 12], and contribute to chemotherapy resistance [13, 14]. TWIST1 has also been shown to be overexpressed in numerous solid tumors [11, 15–19], including aggressive and metastatic forms of breast cancer [20–22]; however, it is not expressed in normal adult tissues. This expression profile coupled with the established role of TWIST1 in numerous metastasis-promoting pathways suggests it is a promising novel target for MBC therapy [23].

The therapeutic use of small interfering RNA (siRNA) for cancer has gained considerable interest since its gene silencing properties were first described [24–26]. Once in the cytoplasm, the siRNA unwinds and associates with Argonaute2, forming an RNA-induced silencing complex (RISC), which leads to sequence-specific mRNA degradation and gene silencing [27]. However, while promising, the development of siRNA therapy has encountered challenges including susceptibility to enzymatic degradation, delivery to target tissues, endosomal escape, immune activation, and off-target effects [28–31]. Effective siRNA delivery (both to the tissue of interest and across the cell membrane) remains one of the main barriers to developing clinically relevant therapies [32, 33].

**Figure 1:**
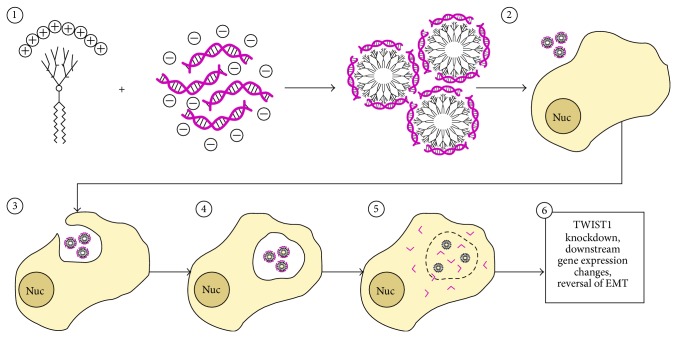
*Mechanism of dendrimer-mediated siRNA delivery and TWIST1 knockdown.* (1) Negatively charged siRNA is electrostatically attracted to positive charges on the YTZ3-15 dendrimer, leading to the formation of 6–8 nm diameter micelles coated with siRNA. (2) These dendriplexes are administered to tumor cells. (3) Dendriplexes are taken up via macropinocytosis. (4) Dendriplexes are trafficked to late endosomes. (5) Due to the proton sponge effect, electrostatic interactions between the dendrimer and siRNA are disrupted and siRNA escapes from the disrupted endosome into the cytosol. (6) Once in the cytosol, siRNA recruits the endogenous RNAi machinery to degrade TWIST1 mRNA. Following TWIST1 knockdown, TWIST1 target gene expression is altered to reduce invasive capacity.

The success of an siRNA-based gene silencing therapeutic approach requires that the siRNA enters the cytoplasm without being degraded [34, 35]. Recent studies have demonstrated that poly(amidoamine) (PAMAM) dendrimers are capable of functional siRNA delivery both* in vitro* and* in vivo* by protecting siRNA (via electrostatic interactions and aggregation) from enzymatic degradation prior to macropinocytosis and eventual release for the endosome (Figure 1) [36, 37]. Recently, a modified third generation amphiphilic PAMAM dendrimer (YTZ3-15) was shown to effectively deliver siRNA and cause gene knockdown* in vivo* via intratumoral (IT) administration [38]. Complexing YTZ3-15 with TWIST1 siRNA may therefore have the potential to allow delivery of potent siRNAs to breast tumor cells to reduce TWIST1-mediated expression of EMT target genes and inhibit metastatic potential.

In the current study, we investigated whether anti-TWIST1 siRNA could be functionally delivered to metastatic breast cancer cells (SUM 1315 cell line) using YTZ3-15. We tested the ability of the YTZ3-15-delivered siRNA to knock down TWIST1, reduce expression of EMT-related target genes, and alter the phenotypic characteristics associated with cancer cell migration/invasion. We also evaluated the tumor-specific delivery capability of YTZ3-15* in vivo* using a mouse breast cancer model.

## 2. Materials and Methods

### 2.1. Cell Culture, Transfection, and Stable Cell Line Production

SUM 1315 breast cancer cells were obtained from ATCC (Manassas, VA). Cells were maintained at 37°C, 5% CO_2_, and 90% humidity in a tissue culture incubator. Media for SUM 1315 cells consisted of a 50-50 mixture of DMEM and F12 media, supplemented with 5 *μ*g/mL insulin, 10 ng/mL EGF, 5% fetal bovine serum (FBS), and 1% penicillin/streptomycin. Cells were passaged using 0.25% trypsin (Genesee Scientific, San Diego, CA) every 2-3 days as they reached confluency. Transient transfection of SUM 1315 cells was performed using Lipofectamine 2000 (Thermo Fisher Scientific, Waltham, MA) or YTZ3-15 (obtained from Dr. Ling Peng, Centre Interdisciplinaire de Nanoscience de Marseille, France). siRNA sequences were as follows: siTwistA-sense, 5′-GGACAAGCUGAGCAAGAUU-3′; siTwistA-antisense, 5′-AAUCUUGCUCAGCUUGUCCUU-3′; siTwistB-sense, 5′-GCGACGAGCUGGACUCCAA-3′; siTwistB-antisense, 5′-UUGGAGUCCAGCUCGUCGCUU-3′. All custom siRNAs were synthesized by IDT (Integrated DNA Technologies, Inc., Skokie, Illinois) and arrived lyophilized and were resuspended in water prior to being reannealed. Two negative control siRNAs were used. siQ (labeled with either AlexaFluor 488 or 647) was AllStars Negative Control siRNA from Qiagen (Valencia, CA), and siCtrl was a nonlabeled control siRNA with the following sequence: siCtrl-sense, 5′-ACUCCAAGAUGGCAAGCUG-3′; siCtrl-antisense, 5′-CAGCUUGCCAUCUUGGAGU-3′. Lipofectamine 2000 was diluted fiftyfold in OptiMEM (Thermo Fisher Scientific; Waltham, MA) and incubated with 10 *μ*L of 10 *μ*M siRNA for 20 min at room temperature. YTZ3-15 was diluted to 1.75 *μ*M in Opti-MEM and mixed with 10 *μ*L of 10 *μ*M siRNA at an N/P ratio of 5, for a final siRNA concentration of 50 nM when applied to cells in 2 mL of media per well in a 6-well plate. Dendrimer complexes (dendriplexes) were incubated 20 min at room temperature which resulted in dendriplex aggregates of roughly 100–200 nm in size (Figure 1) [39]. Incubation of the YTZ3-15 dendriplexes with SUM 1315 cells was done at the tissue culture conditions described previously for up to 7 days with fresh media being added to existing media as needed.

Stable transfections of SUM 1315 cells were performed using lentivirus. To examine the effects of TWIST1 knockdown in SUM 1315 cells without the possible confounding variables of the delivery mechanism itself, we developed cell lines that stably expressed short hairpin RNAs (shRNAs) against TWIST1 (shTwistA, shTwistB), or a scrambled control shRNA (shScram) as a negative control. The mRNA target sequences for shTwistA and shTwistB are identical to the sense sequences of siTwistA and siTwistB (mentioned in the previous paragraph), respectively. The mRNA “target” sequence for shScram was 5′-UUCUCCGAACGUGUCACGU-3′. Cells were either stably transfected with shTwistA, shTwistB, or shScram as described previously [40]. Cells expressing eGFP-firefly luciferase fusion protein (eGFP + luc) were created by transfecting the CMV cassette as described previously [41]. Immortalized human mesenchymal stem cells (hMSCs) were used for* in vivo* experiments as described previously [42].

The YTZ3-15 dendrimer has been described in detail previously [38, 39], but will be briefly summarized here. The chemical formula for each YTZ3-15 dendrimer is C_125_H_247_N_37_O_20_ with a molecular weight of 2586.9448 daltons. They are formed by click chemistry and consist of two lipid tails at one end and a dendron with eight terminal amines on the opposite end. The dendrimer was purified by column chromatography on silica gel with Petroleum ether/EtOAc [38]. These dendrimers spontaneously aggregate to form micelles ranging in size from 100 nm to 200 nm. When in the presence of siRNA, these dendrimers rearrange into smaller (6–8 nm) substructures as part of the larger micelles in order to allow for more electrostatic interactions between the negatively charged siRNA and the positively charged amines on the dendrimer (Figure 1) [39].

### 2.2. Wound Healing Assay

To examine directional cell migration,* in vitro *wound healing assays were performed as described previously [43]. In summary, SUM 1315 cells (parental, eGFP + luc, shScram, shTwistA, and shTwistB) were grown in the conditions described above in Section 2.1 in 6-well tissue culture plates. Once cells reached 80% confluency, a sterile 200 *μ*L pipette tip was used to scratch a line in the monolayer of cells. Images were taken immediately after the scratch and at several time points thereafter using a Nikon TE-2000S microscope and SPOT Advanced software (Diagnostic Instruments, Sterling Heights, MI). Care was taken to always capture images in the same location for each time point. Additionally, SUM 1315 cells were transfected with siQ-, siTwistA-, or siTwistB-YTZ3-15 dendriplexes at a 50 nM final siRNA concentration (1.75 *μ*M YTZ3-15). Cells were incubated with dendriplexes for 24 hours at 37°C, 5% CO_2_, and 90% humidity in a tissue culture incubator; the plates were then scratched with the 200 *μ*L pipette tip and imaged as described above.

### 2.3. Invasion Assay

2.5 × 10^5^ SUM 1315 cells were transfected using YTZ3-15 complexed with siQ, siTwistA, or siTwistB as described above in Section 2.2. After 24 hours incubation, 2.5 × 10^5^ SUM 1315 cells were lifted from the plate with 0.25% trypsin, washed with PBS, and seeded onto transwell inserts (8 *μ*m pore diameter; Millipore, Darmstadt, Germany). Inserts (which nest inside wells in a 24-well tissue culture plate) were precoated with Matrigel (3 mg/mL, 60 *μ*L, diluted with serum-free medium) (BD Biosciences, San Jose, CA), which was allowed to solidify in a tissue culture incubator for 30 minutes prior to the addition of the transfected cells. In order to stimulate cell invasion the top chamber containing the transfected SUM 1315 cells contained 1% FBS (400 *μ*L) while the lower chamber contained 600 *μ*L of complete media with 20% FBS. Cells were permitted to invade for 24 hours in a tissue culture incubator. After this period the Matrigel and any remaining cells in the upper chamber were removed with a cotton-tipped swab. Transwell membranes were then washed twice with PBS and stained with Crystal Violet. Five images of each membrane were taken and cells were counted manually.

### 2.4. Quantitative PCR

Total cellular RNA was isolated using the RNeasy Plus kit (Qiagen, Valencia, CA). RNA quantity and quality were measured and analyzed (with 260/280 nm and 260/230 nm spectra measurements) using a NanoDrop ND-1000 and its associated software (Thermo Fisher Scientific, Waltham, MA). An equal amount of RNA for all conditions was used as a template for cDNA synthesis using the iScript cDNA Synthesis kit with provided random primers (Bio-Rad, Hercules, CA). Quantitative RT-PCR (qPCR) was performed in triplicate using 500 ng/well cDNA and Maxima SYBR Green Master Mix (Thermo Fisher Scientific, Waltham, MA) in 25 *μ*L reactions. Cycling was conducted in a Bio-Rad iQ5 thermal cycler for 40 cycles (95°C, 15 s; 57°C, 60 s; 79°C, 30 s) followed by melt curve analysis. Data were analyzed using Bio-Rad iQ5 software (2^−ΔΔCt^ method, normalized to *β*-Actin). Primers used were TWIST forward #1, 5′-CTATGTGGCTCACGAGCGGCTC-3′; TWIST reverse #1, 5′-CCAGCTCCAGAGTCTCTAGACTGTCC-3′; TWIST forward #2, 5′-TCTTACGAGGAGCTGCAGACGCA-3′; TWIST reverse #2, 5′-ATCTTGGAGTCCAGCTCGTCGCT-3′; N-Cadherin forward, 5′-GGGACAGTTCCTGAGGGATCAA-3′, N-Cadherin reverse, 5′-TGGAGCCTGAGACACGATTCTG-3′, Vimentin forward, 5′-TCGTCACCTTCGTGAATACCAAGA-3′, Vimentin reverse, 5′-CCTCAGGTTCAGGGAGGAAAAGTT-3′, *β*-Actin forward, 5′-CCGCAAAGACCTGTACGCCAAC-3′; *β*-Actin reverse, 5′-CCAGGGCAGTGATCTCCTTCTG-3′.

### 2.5. Western Blot

Cells were seeded at 250,000 cells per well in 6-well tissue culture plates and treated as described in Section 2.1. Cells were pelleted and lysed in RIPA buffer, and protein concentration was determined using a BCA Assay (Thermo Fisher Scientific, Waltham, MA). 30 *μ*g total protein per lane was run on 4% stacking and 10–12% resolving polyacrylamide gels and transferred to Immobilon-P PVDF membrane (Millipore, Billerica, MA) using a Trans-Blot SD Semi-Dry Transfer Cell (Bio-Rad, Hercules, CA). Membranes were blocked with 5% dry milk dissolved in 1X PBS with 0.1% Tween-20. Antibodies were diluted in blocking buffer. Antibodies used were anti-TWIST, TWIST 2c1a (Santa Cruz Biotech, Dallas, TX); anti-*β*-Actin, A1978 (Sigma Aldrich, St. Louis, MO); and Horse Radish Peroxidase (HRP) conjugated anti-mouse secondary antibodies. ECL Plus chemiluminescent substrate (Pierce, Thermo Fisher Scientific, Waltham, MA) and Blue Devil Film (Genesee Scientific, San Diego, CA) were used for capturing images.

### 2.6. Confocal Microscopy

SUM 1315 cells were transfected with dendriplexes comprised of siQ (labeled with AlexaFluor 647) and YTZ3-15 and incubated for 24 hours in a tissue culture incubator. Cells were then treated with LysoTracker Red (Thermo Fisher Scientific, Waltham, MA) according to the manufacturer's protocol. Confocal images were obtained using the Zeiss LSM 700 Confocal Microscope and ZEN 2012 microscopy software (Zeiss AG, Oberkochen, Germany).

### 2.7. Tumor Engraftment

A total of six female NOD.Cg-*Prkdc* ^*scid*^ *Il2rg* ^*tm1Wjl*^/SzJ (NSG) mice (The Jackson Laboratory, Bar Harbor, ME) were used in a pilot study to engraft the SUM 1315 eGFP + luc breast cancer cells (8 weeks old at time of inoculation). The six mice were to be divided into two groups: intratumoral (IT) and intravenous (IV). While under anesthesia (Isoflurane, 2.5–4%), mice received bilateral inoculations of cells into the 4th mammary fat pad. Inoculum for each mammary fat pad consisted of 1 × 10^6^ SUM 1315 eGFP + luc cells together with 2 × 10^5^ hMSCs suspended in 50 *μ*L 3 mg/mL Matrigel. Injections were delivered into the mammary fat pad adjacent to the nipple. Mice were then allowed to recover in a clean cage. Two NSG mice receiving no cells were used as controls.

### 2.8. *In Vivo* Imaging

After tumor cell inoculations, the mice were imaged every two weeks using the Xenogen IVIS 100 biophotonic imaging system (STTARR, Toronto, Ontario, Canada) to monitor tumor growth. To obtain* in vivo* images, mice were given a 200 *μ*L intraperitoneal (IP) injection of 25 mg/mL D-Luciferin (PerkinElmer, Waltham, MA). After a 10-minute waiting period, animals were anesthetized using Isoflurane (2–5%) and placed in a black box in the biophotonic imager. Bioluminescent images were captured over a period of one minute. Once tumors had reached 0.5–0.75 cm by caliper measurement, three mice were given a single intravenous (IV) injection of the YTZ3-15 + siQ dendriplex (15 *μ*L of 240 *μ*M YTZ3-15 and 10 *μ*L of 10 *μ*M siQ) diluted in 200 *μ*L PBS. A separate group of three animals was given intratumoral injections of the YTZ3-15 + siQ dendriplex (15 *μ*L of 240 *μ*M YTZ3-15 and 10 *μ*L of 10 *μ*M siQ) diluted in 100 *μ*L PBS. After the injections, animals underwent* in vivo* fluorescent imaging using the IVIS 100 (Cy5.5 filter). Images were captured at 5, 10, 15, and 240 minutes after injection of the dendriplexes. After the final time point (4 hours), all animals were euthanized and tissues were collected. Tumors, spleen, kidney, and liver from each animal were imaged* ex vivo* using the IVIS 100 to detect the AlexaFluor 647-labeled siQ without the hindrance of the skin and fur. Two NSG mice receiving no cells were used as controls for* in vivo* imaging.

### 2.9. Statistics and Replications

Wound healing assays were repeated three times as were the Western blot analyses. Invasion assay was repeated twice with identical conditions. Five images were captured for each invasion assay condition and the numbers of cells were counted manually and standard deviations were calculated using Excel (Microsoft, Redman, WA). qPCR experiments were done in triplicate and analyzed using the 2^−ΔΔCt^ method in the Bio-Rad iQ5 software. Three animals per group (along with two control animals) were used for biodistribution purposes only.

## 3. Results and Discussion

### 3.1. Stable TWIST1 Knockdown in SUM 1315 Cells

**Figure 2:**
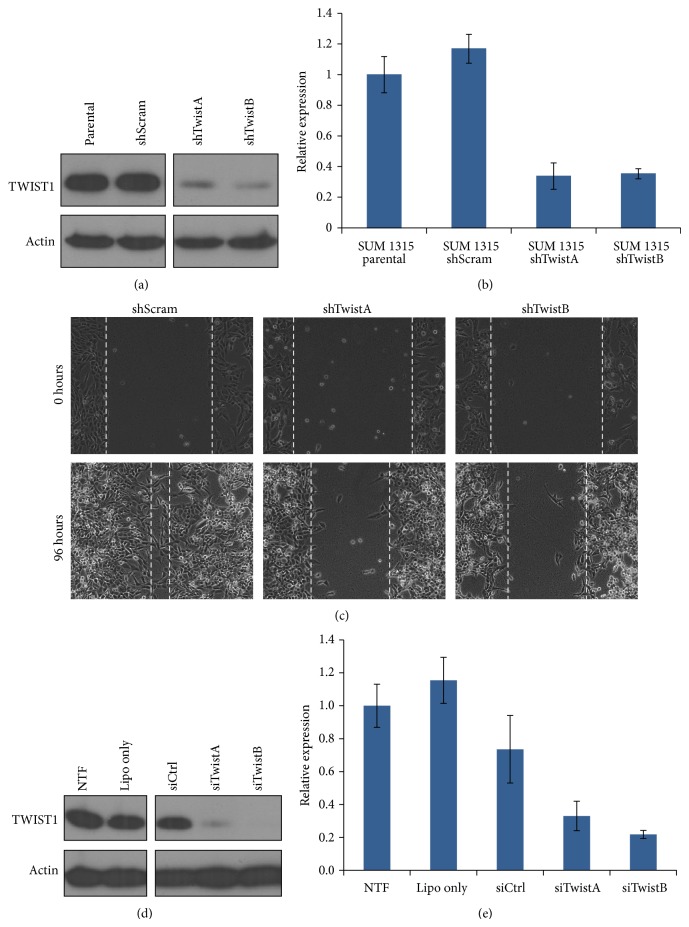
*Stable and transient RNA-mediated TWIST1 knockdown in SUM 1315.* (a) Western blotting demonstrated robust TWIST1 knockdown in both shTwistA and shTwistB lines. (b) qPCR confirmed TWIST1 knockdown at the mRNA level for both stable knockdown lines. Error bars represent standard deviation. (c) SUM 1315 cells expressing shTwistA or shTwistB exhibited decreased directional migration compared to those expressing shScram control in wound healing assays. Dashed lines indicate migratory front and were added manually. Images shown are representative data from experiments performed in triplicate. (d) Western blot demonstrated substantial TWIST1 knockdown in SUM 1315 cells transfected with siTwistA and siTwistB using Lipofectamine 2000 when compared to nontransfected (NTF), Lipofectamine 2000 alone (Lipo only), or control siRNA (siCtrl). (e) qPCR results mirrored those seen in the Western blot.

The relationship between TWIST1 expression and EMT has been established for breast cancer [44]. To examine the effects of TWIST1 knockdown in SUM 1315 cells without the possible confounding variables that a delivery mechanism may cause, we developed cell lines that stably expressed shRNAs against TWIST1 (shTwistA and shTwistB) and a scrambled shRNA (shScram) as a negative control. TWIST1 expression in the SUM 1315 shTwistA and shTwistB cell lines demonstrated excellent knockdown of TWIST1 compared to the parental line and the shScram line (Figures 2(a) and 2(b)).

To test the effect of TWIST1 knockdown on cell migration, we performed a wound healing assay. Our results demonstrated that the SUM 1315 shTwistA and shTwistB cell lines had reduced directional migratory abilities compared to the SUM 1315 shScram cell line (Figure 2(c)). Taken together, these data suggest not only that shTwistA and shTwistB significantly knock down expression of TWIST1 in SUM 1315 cells, but also that the downregulation of TWIST1 results in a phenotypic change consistent with diminished migratory ability.

### 3.2. siRNA-Mediated TWIST1 Knockdown in SUM 1315 Cells

**Figure 3:**
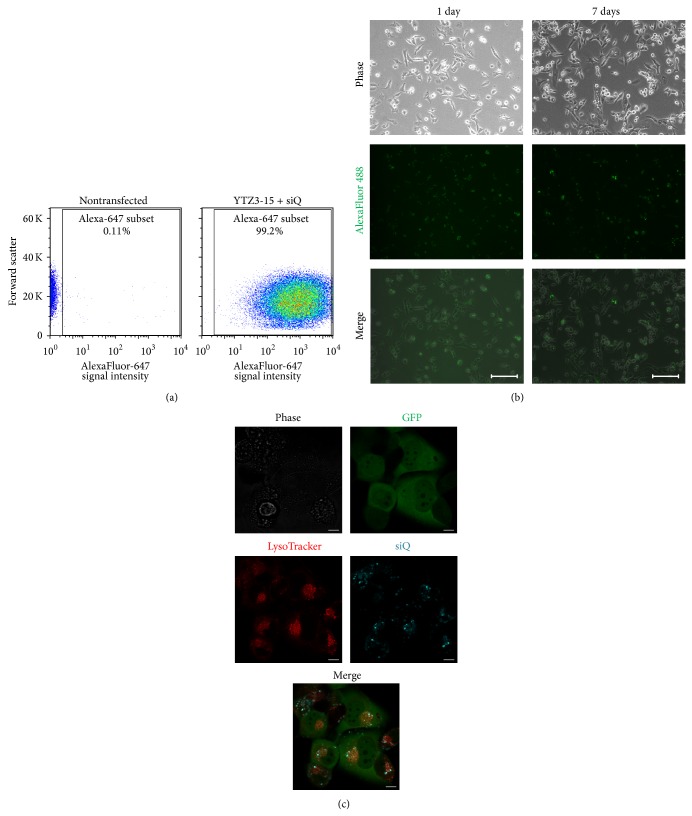
*YTZ3-15 effectively delivers siRNA to SUM 1315 cells.* (a) Left: nontransfected SUM 1315 cells had low background fluorescence. Right: more than 99% of YTZ3-15 transfected cells were positive for AlexaFluor 647-labeled siQ. (b) Fluorescent microscopy revealed that AlexaFluor 488-labeled siQ was taken up into cells within one day, and AlexaFluor signal was still detectable in cells at seven days after transfection. (c) Confocal images of SUM 1315 cells stably expressing eGFP + luc and transiently transfected with AlexaFluor 647-labeled siQ using YTZ3-15. LysoTracker dye revealed that siQ primarily colocalized with mid to late endosomes after 24-hour incubation with YTZ3-15 siRNA dendriplexes.

The SUM 1315 shRNA results described above demonstrate not only a significant reduction in the amount of TWIST1 expression, but also a phenotypic change in cell migration, suggesting that these shRNA sequences were effective in knocking down TWIST1 expression. We thus designed siRNA sequences (siTwistA and siTwistB) based on these shRNA sequences. To test the efficacy of siTwistA and siTwistB, SUM 1315 cells were transfected using Lipofectamine 2000. Transfection with either siTwistA or siTwistB resulted in knockdown of TWIST1, with siTwistB giving slightly more knockdown than siTwistA at both the protein and mRNA levels (Figures 2(d) and 2(e)). Next, we tested the delivery of siRNA into SUM 1315 cells using the YTZ3-15 dendrimer. Cellular uptake of AlexaFluor 647-labeled siQ (acting as a surrogate for unlabeled siTwistA and siTwistB) was greater than 90% after 24 hours, as measured by flow cytometry and fluorescent microscopy (Figures 3(a) and 3(b)). The presence of siQ in cells transfected using YTZ3-15 dendriplexes was confirmed as far out as 7 days from the time of transfection (Figure 3(b)). These findings confirm previous work [38] performed with this PAMAM dendrimer and demonstrate its ability to safely deliver siRNA across the cell membrane, because we did not appreciate any increase in cell death. Cellular uptake using YTZ3-15 + siQ was comparable when tested in other cell lines including other breast, ovarian, uterine, and prostate cancer cell lines (data not shown).

While uptake of the dendriplexes can be appreciated with fluorescent microscopy and flow cytometry, these methods do not indicate the location of the siRNA within the cell. To examine this, we used LysoTracker Red (dye taken up into acidic organelles) to show where siQ is localized. Our results show that much of the siQ signal colocalizes with the mid to late endosome in the SUM 1315 eGFP + luc cell line (Figure 3(c)). siRNA localization to these organelles is desirable to take advantage of the “proton sponge effect,” which is thought to be essential for siRNA release [45, 46].

**Figure 4:**
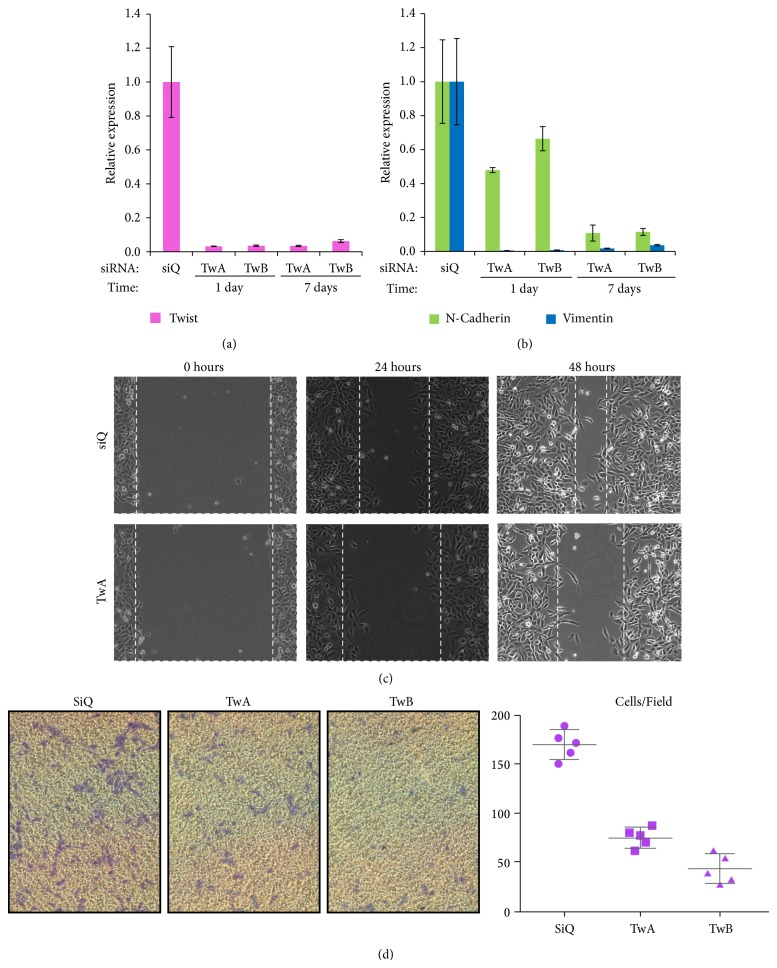
*TWIST1 knockdown following YTZ3-15 delivery of siTwist decreases cell motility and downstream EMT marker expression.* (a) Compared to siQ control (at seven days), siTwistA (TwA) and siTwistB (TwB) delivered via YTZ3-15 produced >90% TWIST1 knockdown at the mRNA level. Knockdown lasted seven days after transfection. (b) Compared to siQ control (at seven days), TwA and TwB delivered via YTZ3-15 produced knockdown of the TWIST1 targets N-Cadherin and Vimentin. N-Cadherin mRNA levels decreased by >40% after one day, and by approximately 90% after seven days. Vimentin mRNA was nearly undetectable after one day, and remained at <10% after seven days. (c) YTZ3-15 transfection of siTwistA decreased directional migration compared to siQ transfected cells (control) in wound healing assays. Dashed lines indicate migratory front and were placed manually. Images shown are representative data from experiments performed in triplicate. (d) Left: YTZ3-15 transfection of TwA or TwB resulted in >50% decrease in invasion of SUM 1315 cells through Matrigel. Cells were allowed to migrate for one day, following one day incubation with YTZ3-15-siRNA dendriplexes. Five fields per condition were imaged (representative images shown). Right: quantification of image data. Bars represent mean and standard deviation of five fields per condition.

After confirming the function of siTwistA and siTwistB with Lipofectamine 2000 and the cellular uptake of siQ using YTZ3-15, we tested siTwistA and siTwistB with YTZ3-15-based delivery. TWIST1 levels were measured using qPCR and found to be significantly reduced at 24 hours and one week after transfection (Figure 4(a)). Two EMT-related TWIST1 target genes (Vimentin and N-Cadherin) also showed reduced mRNA expression. Vimentin and N-Cadherin were both substantially reduced at the 24-hour time point; however, Vimentin showed a slight return at the one week time point, whereas N-Cadherin continued to decrease (Figure 4(b)). While reduced expression of these genes was noted, renewed expression of the epithelial marker E-Cadherin was not observed (data not shown). This is a noted difference from previous studies [4]. The possible causes for this discrepancy are the different cell lines used between previous studies and ours, and that E-Cadherin is not entirely controlled by TWIST1 [19, 47]. Reduced expression of these EMT-related genes is a positive indication that migration and invasion would be hindered.

Next, we performed a wound healing assay to validate that YTZ3-15-delivered siRNA against TWIST1 not only reduces the expression of TWIST1 and its target genes, but also inhibits the migratory action of SUM 1315 cells. This assay demonstrated decreased directional migration of SUM 1315 cells transfected with siTwistA (Figure 4(c)).

The EMT process consists of migration and invasion, and TWIST1 is a major factor in allowing cancer cells to infiltrate surrounding tissues, blood vessels, and the lymphatic system [40, 48]. To investigate whether the invasive phenotype is reduced following siRNA-mediated TWIST1 knockdown, we performed a transwell invasion assay. Results indicated that the YTZ3-15 + siRNA-treated cells had diminished abilities to invade the Matrigel matrix and pass through the porous membrane, thus indicating a reduction in the invasive phenotype (Figure 4(d)). TWIST1 overexpression is associated with cancers that are more metastatic and therefore invasive [22], and these data show that TWIST1 silencing following dendriplex delivery of siRNA decreases metastatic potential. This in turn suggests that as a therapeutic approach for patients with MBC, this delivery method and target could have a significant impact on improving survival and outcomes for MBC patients if preclinical and clinical trials show similar results.

### 3.3. *In Vivo* Distribution of PAMAM Dendrimers

**Figure 5:**
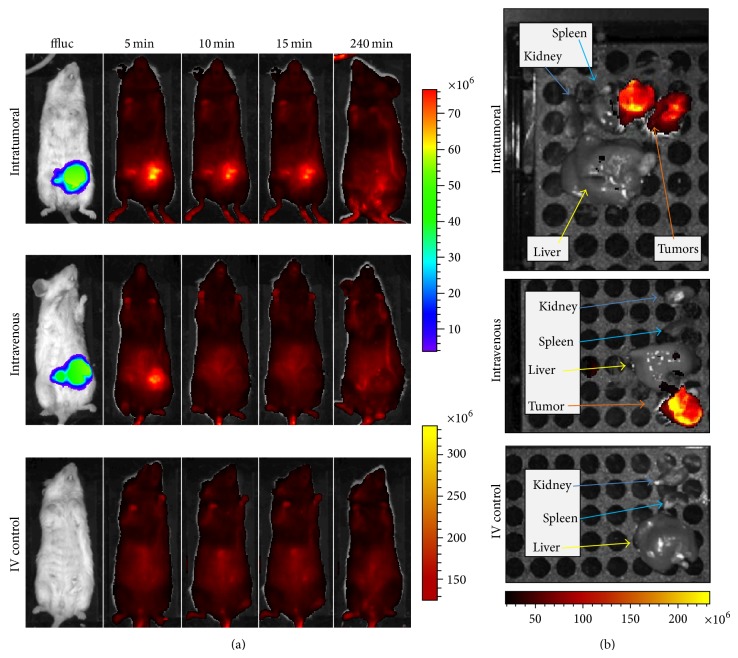
*YTZ3-15 concentrates in orthotopic breast cancer tumors in vivo*. (a) Representative animals from the mice that received YTZ3-15 + siQ via intratumoral (IT) and intravenous (IV) injections. Control animals received IV injections of the dendriplexes but had no tumors. Mice receiving IT injections showed accumulation of siQ lasting at least 15 minutes post injection, whereas mice receiving IV injections showed little accumulation after 5 minutes. Control animals do not show accumulation of siQ due to the absence of tumors. (b)* Ex vivo* imaging of spleen, kidney, liver, and tumors (where applicable) from the three animals shown in Figure 5(a), demonstrating concentration of YTZ3-15 + siQ dendriplexes in the tumors but not in other organs. Images were obtained 240 mins after the administration (IT or IV) of YTZ3-15 + siQ. The units for the scale bars in this figure are photons/sec/cm^2^/steradian.

*In vivo* studies were completed to determine the optimum delivery route (IV versus IT) of siQ using YTZ3-15. Five minutes after the IV or IT injection of the YTZ3-15 + siQ dendriplex, a bright signal was noted at the site of the tumor (Figure 5(a)). The signal at the tumor site continued to be evident in mice that received IT injections at 10, 15, and 240 minutes, whereas no signal was seen at the tumor site after 5 minutes in mice that received IV injections (Figure 5(a)). However,* ex vivo* imaging of tumors, spleen, liver, and kidneys after 240 minutes revealed a robust AlexaFluor 647 signal in the tumors but little to no signal in other examined organs (Figure 5(b)). This* ex vivo* tumor-centric signal was evident for all mice, regardless of the route of administration (IV versus IT).

While the YTZ3-15 dendrimer does not have any inherent tumor-targeting capabilities, results from our* in vivo *studies demonstrate that these dendriplexes do accumulate preferentially in the orthotopic breast cancer tumors. It is possible that localization to the tumor is due to the enhanced permeability and retention (EPR) effect, which has been seen with other PAMAM dendrimers and nanoparticle delivery vehicles [49–51]. The inherent leakiness of tumor vasculature coupled with minimal lymphatic drainage results in particles becoming trapped and consequently concentrated in the tumor environment. This effect is magnified as the tumor enlarges and promotes angiogenesis, which may explain why siQ concentration was noted only after orthotopic tumors reached 0.5 × 0.5 cm in size (data not shown).

In addition to our promising results, there are other important reasons to focus on the knockdown of TWIST1 in cancer cells. TWIST1 plays an essential role in early embryonic development as evidenced in mice and humans with heterozygous gene mutations, where both have craniofacial abnormalities (Saethre-Chotzen syndrome in humans) [52, 53]. Additionally, TWIST1 knockout mice are embryonic lethal [54, 55]. Given that TWIST1 is crucial in early development it is not surprising that it maintains the CSC phenotype [5–7, 10, 56]. The CSC phenotype is associated with an undifferentiated cellular morphology, increased mobility, self-renewal, resistance to apoptosis, and chemoresistance [7]; thus silencing of TWIST1 may aid in weakening those cells that are most resilient to current therapeutic modalities.

A TWIST1 siRNA therapeutic approach to assist in the treatment of MBC is also attractive because it could complement and augment current treatment regimens. For example, it is known that TWIST1 overexpression in breast cancer is associated with a poorer prognosis partly due to downregulation of estrogen receptor *α* (ER-*α*) [13, 14, 21]; a reduction in ER-*α* leads to a diminished sensitivity to hormone therapies. Furthermore, TWIST1 expression is associated with resistance to commonly used chemotherapy agents in many human carcinomas [15, 57], and it has been demonstrated that a reduction of TWIST1 can resensitize tumor cells to chemotherapy [58]. TWIST1 is also an intriguing therapeutic target because for almost all adult tissues TWIST1 expression is nonexistent [8, 59]. Therefore, if a TWIST1-specific therapy could be delivered, the side effects on nontumor tissue would be minimal because there is no TWIST1 to knock down. Taken together, these data and observations suggest that siRNA-based knockdown of TWIST1 could be used in conjunction with hormonal therapy or chemotherapy to achieve a synergistic effect. Such a combined approach (chemotherapy plus anti-TWIST1 siRNA) is currently being explored by our laboratory and others using various types of nanoparticles that allow for simultaneous delivery into breast cancer cells [60–62].

## 4. Conclusions

Our studies demonstrate successful delivery and utilization of two siRNAs against TWIST1. Delivery was realized using a modified third generation PAMAM dendrimer and resulted in significant knockdown of TWIST1 and other EMT-related target genes* in vitro*. TWIST1 knockdown resulted in a reduction in cellular migration and invasion as has been observed previously [9, 11, 40, 48, 63]. Finally, delivery of an siRNA by YTZ3-15 was shown to have a specific concentrating ability in orthotopic tumors in a TNBC mouse model.

These data add to the growing evidence that TWIST1 is an important and potentially clinically significant therapeutic target for the treatment of MBC as well as other solid tumor cancers [23, 64, 65]. While TWIST1 knockdown via PAMAM dendrimer-delivered siRNA could not reasonably be used as a sole means of treatment for MBC, it could serve as a valuable tool and adjuvant therapy to reduce migration/invasion, chemoresistance, and antiapoptotic tendencies associated with aggressive tumors. Novel results from this study serve to validate a multimodal approach to cancer treatment by focusing on a transcription factor associated with breast cancer tumor types that have minimal treatment options (e.g., TNBC). Furthermore, these data support further investigations (both* in vitro* and* in vivo*) into the use of siRNA coupled with nanoparticles to treat malignant breast cancer by knocking down TWIST1 and its associated EMT targets.
